# The impact of a discrepancy between actual and preferred living arrangements on life satisfaction among the elderly in China

**DOI:** 10.6061/clinics/2015(09)05

**Published:** 2015-09

**Authors:** Jinqun Guan, Hui Li, Hong Sun, Ting Wang, Weiping Wu

**Affiliations:** I Chinese PLA General Hospital, Department of Nanlou, Clinical Division of Neurology, ChinaChina; II First Hospital of Hebei Medical University, Department of Neurosurgery, ChinaChina

**Keywords:** Life Satisfaction, Actual Living Arrangement, Preferred Living Arrangement, Discrepancy

## Abstract

**OBJECTIVES:**

To explore the effects of a discrepancy between actual and preferred living arrangements on the relationship between living arrangements and life satisfaction among the elderly in China.

**METHODS:**

Secondary analysis of the 2005 dataset of the Chinese Longitudinal Healthy Longevity Survey was performed. A binary logistic regression model was used to analyze the relationship between life satisfaction and living arrangements.

**RESULTS:**

Among those with concordant actual and preferred living arrangements, living in a nursing home increased the likelihood of life satisfaction, whereas living alone and living with a spouse decreased the likelihood of life satisfaction compared to living with the next generation and a spouse. Among those with discordant living arrangements, there were no differences in life satisfaction between the various living arrangements, except that living with a spouse increased life satisfaction compared to living with the next generation and a spouse.

**CONCLUSIONS:**

A discrepancy between actual and preferred living arrangements modifies the relationship between life satisfaction and actual living arrangement. Living in a nursing home is a good option for Chinese elder care only if the older individual emotionally accepts it. Living alone or with a spouse is not a good arrangement for elder care, even though it is often preferred by the elderly. Those with discordant living arrangements are more satisfied living with their spouses.

## INTRODUCTION

Like many other Asian cultures, Chinese culture emphasizes filial piety. It is considered the responsibility of a son to respect and care for his parents. Co-residence with the next generation is the traditional and most common living arrangement of the elderly in China. However, China is currently experiencing an aging boom. The proportion of the population aged 60 or older in China has dramatically increased, from just over 7% in 1953 to more than 10% in 2000 1 and to 14.3% in 2012 2. Along with the rapid aging of the population, the Chinese family planning policy has also led to fewer family members taking care of the elderly through co-residency, and the proportion of Chinese elderly living under this traditional arrangement has decreased, especially after 1990. The percentage of men aged 65 and above who co-resided with the next generation was 67.9, 67.6, and 59.9% in 1982, 1990, and 2000, respectively; the corresponding percentage of women aged 65 and above was 73.6, 74 and 68.7%, respectively 3. An increasing number of Chinese elderly live in nontraditional households, such as living only with a spouse, living alone, or living in nursing homes.

Living arrangements are important to the elderly because “various household structures make very different demands on the adults in them and offer very different levels and types of resources” 4. Previous studies have shown that living arrangements are related to life satisfaction in the elderly. Studies have shown that in the Asian culture, in-home co-residency with the next generation is results in greater life satisfaction in the elderly compared to other living arrangements 5-6. However, these studies did not consider the living arrangement preference of the elderly and assumed that the actual living arrangement represented the preferred living arrangement. In fact, the living arrangements of the elderly are complicated. The actual living arrangement can result from either active acceptance, as when the elderly individual chooses the arrangement, or passive acceptance, as occurs when the arrangement is based on practical constraints. An elderly person who prefers to live alone because he wants to have his own independent life and therefore actually chooses to live alone is an example of active acceptance. An elderly person who lives with his married son not because he prefers to but because his son does not have his own apartment and needs to live with his father is an example of passive acceptance. Overall, a discrepancy between actual and preferred living arrangements is common in China. Logan and Bian 7 found that one-third of elderly parents in urban China in the late 1980s did not have a living situation that they considered ideal.

In the self-discrepancy theory, Higgins 8 postulates that humans are motivated to reach a condition in which the actual self matches the ideal self. The actual self represents the attributes that people believe they actually possess (e.g., “I am living in a nursing home now”), whereas the ideal self represents the attributes that people would ideally possess (e.g., “Hopefully I can live together with my son”). When people believe they have lost or will never reach their ideal self, they feel disappointed. With respect to living arrangements, the elderly will attempt to maintain coherence between their actual and ideal living arrangements. A discrepancy between their actual and preferred living arrangements can make the elderly experience negative emotions and can influence the true relationship between actual living arrangements and life satisfaction. In this paper, data from the 2005 Chinese Longitudinal Healthy Longevity Survey (CLHLS) were analyzed to explore impact of a discrepancy between actual and preferred living arrangements on the relationship between living arrangements and life satisfaction in the Chinese elderly.

## METHODS

### Data Sources

The dataset, which was downloaded from the Public Health Scientific Data Sharing Service, came from the 2005 CLHLS. Only elderly people who expressed their living arrangement preferences clearly were included in the study. Those who did not have a preference were excluded. The final sample size was 13,490 of the original 15,638.

### Variables and measures

The key dependent variable was the self-evaluated life satisfaction, which was a two-category variable. It was derived from a single question, “How do you feel about your life?” The following five levels of responses were possible: very good, good, average, bad, and very bad. We coded “very good” and “good” as 1, to represent “satisfaction”, and we coded “average”, “bad” and “very bad” as 0, to represent “fair/unsatisfied”.

Actual living arrangement was the key independent variable and was classified into five mutually exclusive categories: living with the next generation and a spouse (the next generation included adults in the next generation, grandchildren in the next generation, and great-grandchildren in the next generation), living with the next generation (no spouse), living with a spouse (without the next generation), living alone, and living in a nursing home.

The variable “discrepancy between actual and preferred living arrangement” was derived from the concordance between the actual and preferred living arrangement. In these data, the preferred living arrangement included three choices: co-residing with the next generation, living alone/with a spouse, and living in a nursing home. The discrepancy between the actual and preferred living arrangement, which was a category variable, was coded 1 to represent discordance and 0 to represent concordance.

Five sets of covariates were included in this study. The first set consisted of demographic variables, including ① age and ② gender; male was coded as 0, and female was coded as 1.

The second set of covariates included the following socioeconomic variables: ① Education: no education was coded as 0, and at least one year of education was coded as 1; ② Residence: rural was coded as 0, and city/town was coded as 1; ③ Self-rated economic status: the participants were asked, “How do you rate your economic status relative to that of other local people?” Five levels of response were possible: very rich, rich, average, poor, and very poor. This variable was treated as a three-level variable by coding poor and very poor as 1, average as 2, and rich and very rich as 3.

The third set of covariates included the following physical health status variables: ① Self-rated health: this was assessed using a single question, “In general, would you say your health is very good, good, fair, poor, or very poor?” This variable was treated as a three-level variable by coding poor and very poor as 1, fair as 2, and good and very good as 3. ② Activities of Daily Living (ADL):there were six ADL items in this study, including bathing, dressing, indoor transferring, toileting, incontinence, and eating. A binary variable was created. If the respondents needed assistance in any of the six ADL items, they were coded as 1 to represent disabled. If they did not need assistance, they were coded as 0 to represent non-disabled. ③ Cognitive status: this was measured using the Chinese version of the MMSE, which includes orientation, registration, attention, calculation, recall, and language, with scores ranging from 0 to 23 9. We treated this variable as a continuous variable, and a higher score represented better cognitive status.

The fourth set of covariates included social support variables. The following three questions were used to assess social support: “Who do you usually talk to frequently in daily life?”, “Who do you talk to first when you need to share your thoughts?”, and “Who do you ask for help when you have a problem/difficulties?” Again, a binary variable was created. If the respondents answered any of the three questions by saying that they had no one to talk to or to help them, they were coded as 0 to represent poor social support. The other responses were coded as 1 to represent good social support.

The last set of covariates included psychological resources for well-being. A total of 7 items were used to measure access to psychological resources, including resources for positive well-being (4 items) and to prevent negative well-being (3 items). For each positive well-being question, the following five levels of responses were possible: never 1, seldom 2, sometimes 3, often 4, and always 5. Five levels of responses were also possible for each negative well-being question: never 5, seldom 4, sometimes 3, often 2, and always 1. We treated this variable as a continuous variable.

### Statistical analysis

The data are described by frequencies, percentages, means, and standard deviations. A binary logistic regression model that was based on previous knowledge was used to analyze the relationship between life satisfaction and living arrangements. *P*-values less than 0.05 were considered statistically significant. All of the statistical analyses were performed using IBM SPSS Statistics 22.0 software. The logistic regression model used was as follows:

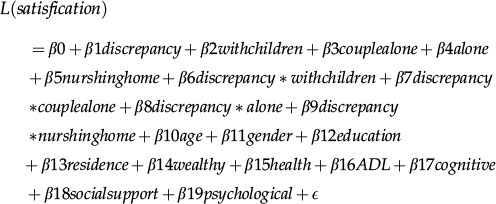


**Note:** Y (1=satisfaction, 0=not satisfaction), discrepancy (1=discordance, 0=concordance), with next generation (1=with next generation, 0=with next generation and spouse), couple alone (1=couple alone, 0=with next generation and spouse), alone (1=alone, 0=with next generation and spouse), and nursing home (1=nursing home, 0=with next generation and spouse).

## RESULTS

The characteristics of the subjects in this study are summarized in Table 1. A total of 2,305 elderly adults from the dataset reported a discrepancy between their actual and preferred living arrangements, resulting in a rate of discrepancy of 17.2%. Among the total population, 61.3% reported being satisfied with their lives. Among those with concordance and discordance, 63.1% and 53.0%, respectively, reported life satisfaction. Among those living with the next generation and a spouse, with the next generation, alone with a spouse, and in a nursing home, 60.2%, 65.6%, 58.9%, 49.4% and 72.4%, respectively, reported life satisfaction. The reported life satisfactions among the respondents in the different living arrangements, stratified by discrepancy between the actual and preferred living arrangements, are shown in Table 2.

After controlling for age, gender, education, residence, self-rated economic status, self-rated health, ADL, cognitive status, social support and psychological resources for well-being, the adjusted ORs for the relationship between life satisfaction and living arrangement among those with concordant and discordant living arrangements are shown in Table 3. Among those with concordance between their actual and preferred living arrangements, living in a nursing home was associated with an increased likelihood of life satisfaction (OR: 2.70, 1.74∼4.21), whereas living alone (OR: 0.62, 0.53∼0.73) and with a spouse (OR: 0.79, 0.68∼0.92, *p*<0.05) were associated with a decreased likelihood of life satisfaction compared to living with the next generation and a spouse. Among those with a discordant arrangement, there was no difference in life satisfaction among all of the living arrangements, except that increased life satisfaction was reported by respondents living with a spouse (compared with living with the next generation and a spouse, indicating that living with a spouse could significantly increase life satisfaction, OR: 1.45, 1.04∼2.01, *p*<0.05).

## DISCUSSION

There are several versions of the CLHLS, namely the 1998, 2000, 2002, 2005, 2008 and 2011 versions 10,11. Accordingly, each of these datasets could be used to perform a study such as the current one. We chose to use the CLHLS 2005 dataset because the 2008 and 2011 datasets were significantly smaller than the 2005 dataset. The reduced size of later versions of the database may be because the CLHLS has longitudinally tracked the very elderly, and over time, a large number of samples are lost due to natural causes. Because the size of the dataset would significantly impact the validity of the data in our study, we chose to use the 2005 CLHLS dataset. Although there have been a few publications based on this dataset 11,12, the focus of our study differed from those of the published studies; moreover, our study provided the first demonstration that a discrepancy between actual and preferred living arrangements is a modulating factor for life satisfaction under different living conditions in the elderly Chinese.

In this study, we found that 2,305 (17.2%) of the elderly adults experienced a discrepancy between their actual and preferred living arrangements, which is lower than the one-third ratio published by Logan and Bian 7. The two study samples had very different age distributions, which may explain this difference. The median age of the respondents in the study of Logan and Bian was 67, which is much lower than the average age of 85.37 in our study. Some individuals who cannot maintain concordance between their actual and preferred living arrangements may try to correct this discrepancy by changing either the reality of their arrangement or their preference. When faced with practical constraints, some may be able to change their values to accommodate situations that limit their options. This means that the ideal can be changed by the reality. If elderly adults cannot maintain concordance between their preferred and actual living arrangements, some may accept their actual living arrangement as the ideal in order to re-establish concordance. The older the individual, the longer they have to adjust, which may be why the much older population in our study had a lower rate of discrepancy between actual and preferred living arrangements than the population studied by Logan and Bian 7.

Even among the very old individuals in this study, the discrepancy rate was still not particularly high (17.2%). Conflicting beliefs about one's living arrangements are believed to trigger emotional distress 8. Compared to elderly adults with concordant living arrangements, it is much more difficult for elderly adults with discordant arrangements to enjoy their actual living arrangements. Thus, the relationships between actual living arrangements and life satisfaction could completely differ between those with concordant situations and those with discordant arrangements. Table 2 shows that the percentage of elderly adults with concordant situations who reported life satisfaction was very different from the percentage of elderly adults with discordant situations. Table 3 shows that among those with concordant arrangements, compared to living with the next generation and a spouse, living in a nursing home (OR: 2.70, 1.74∼4.21) significantly increases the likelihood of life satisfaction, whereas living alone (OR: 0.79, 0.68∼0.92) and living with a spouse (OR: 0.79, 0.68∼0.92) significantly decreased the likelihood of life satisfaction. However, among those with discordant arrangements, compared to living with the next generation and a spouse, living only with a spouse significantly increased the likelihood of life satisfaction. These results indicate that when exploring the relationship between life satisfaction and actual living arrangement, discrepancy between the actual and preferred living arrangements acts as an effect modifier, which is “the variable across which the effect-measure varies” 13. Therefore, when comparing life satisfaction across different living arrangements, it is important to consider discrepancy between actual and preferred living arrangements.

When the data were stratified according to discrepancy between actual and preferred living arrangements, the relationships between life satisfaction and living arrangements provided considerable interesting information. First, living in a nursing home is a promising way to care for elderly Chinese adults. Table 2 shows that among those with concordant living situations, life satisfaction was the highest for those living in a nursing home (79.5%). However, among those with discordant arrangements, the reported life satisfaction of those living in a nursing home decreased sharply to 49.4%. Table 3 shows that compared to living with the next generation and a spouse, living in a nursing home could significantly increase the likelihood of life satisfaction among those with concordant living arrangements (OR: 2.70, 1.74∼4.21); however, this effect disappeared among those with discordant arrangements (OR: 1.46, 0.74∼2.85), which indicates that living in a nursing home can provide a high quality of life for elderly Chinese adults if they accept it whole-heartedly. Although the number of respondents who lived in a nursing home was not large in this study (n=370), the result still confirms, to some extent, that organized elder care is a promising way for the elderly to enjoy later life in China.

However, two challenges must be overcome for elderly Chinese adults to enjoy life in a nursing home. One is psychological resistance to living in a nursing home. In this study, only 463 (3.5%) of the elderly adults preferred to live in a nursing home, whereas 8,026 (59.8%) of the elderly adults preferred to co-reside with the next generation. The majority of elderly Chinese people still prefer to live in the communities they are familiar with and enjoy life with their families. The other challenge is that there are currently few elder care organizations in China. In 2012, there were 193.9 million elderly people aged 60 years and older but only 42 thousand elder care organizations; altogether, these organizations provide 3.810 million beds 2. This means that on average, only 1.96% of elderly adults had the opportunity to live in elder care organizations in 2012. Now, it is time for the Chinese government to improve the acceptance rate of elderly Chinese adults into nursing homes and to expand the number of professional elder-care organizations in China.

Second, the so-called “empty nest” elderly adults are those who live alone or with only a spouse because they do not have children or because their children have already left home 14. In recent years, the population of “empty nest” elderly has been growing in China 15-17. A relatively high percentage of the elderly respondents (36.7%) in our study expressed that they preferred to lie alone or with a spouse, but we found that among those with concordant living situations, both living alone and with a spouse decreased the likelihood of life satisfaction compared to living with the next generation and a spouse (Table 3). This result suggests that even though elderly adults may report a preference to live alone or with a spouse, living as empty nesters does not improve life satisfaction in the elderly.

Third, the life satisfaction of those living with a spouse, which can be considered a special type of empty nest living situation, was very different that of those living alone, even though neither arrangement involves co-habitation with the next generation. In our study, among the total population, the life satisfaction rate among those who lived with a spouse (58.9%) was much higher than the rate among those who lived alone (49.4%) (Table 1). Among those with concordant living arrangements, even though both living alone and living with a spouse decreased the likelihood of life satisfaction compared to living with the next generation and a spouse, the impact of the discrepancy on life satisfaction varied in degree. The OR of living with a spouse VS. with the next generation and a spouse [0.79 (0.68∼0.92)] was higher than that of living alone VS. with the next generation and a spouse [0.62 (0.53∼0.73)], indicating that those who live with a spouse are more likely to experience life satisfaction than those who live alone. It is easy to understand this result because a spouse can provide essential psychological support for the elderly. We can propose that living with a spouse is a type of complete family, but living alone is not. When comparing life satisfaction among those with discordant living arrangements, we found that out of all of the living arrangements, the life satisfaction rate was highest among those living with a spouse (56.9%) (Table 2). After controlling for all of the confounds, the binary logistic regression showed that living with the next generation and a spouse had the same effect on the life satisfaction of the elderly as all of the other living arrangements except living with a spouse (Table 3), indicating that living with a spouse is the best living arrangement for those with discordance.

In our study, individuals who would prefer not to co-reside with the next generation but who did live with them represent a type of discordance. Some elderly individuals may not want to live with the next generation because they want to have their own independent life and avoid conflict with the next generation 7. Previous literature has shown that there is a growing generation gap in attitudes and behaviors in Asian families 18. The beliefs and behaviors of the next generation are very different from those of their elderly parents; thus, conflicts between the generations are common and are not easy to reconcile. Elderly adults may not prefer to co-reside with the next generation because of a strained relationship, but they may have to live with them due to practical constraints; for example, the members of the next generation may not have their own house. In this situation, co-residency is no longer the desired way for the elderly to enjoy their lives and becomes just another living arrangement. For this reason, the life satisfaction reported by those living with the next generation and a spouse did not differ from that reported by those with other living arrangements. For those who live with only a spouse and who do not like this arrangement, although the potential problems associated with old age may be a negative aspect, the lack of conflict with the next generation may also be positive, and the relationship between the elderly individual and their spouse is often good. Thus, living with a spouse becomes an outstanding living arrangement for those with discordance.

## Figures and Tables

**Table 1:** Characteristics of the study subjects (n=13,490).

	Frequency (%)
**Gender**	
Male	5,943 (44.1)
Female	7,547 (55.9)
**Education**	
Not educated	8,051 (59.9)
Educated	
**Residence**	
Rural	7,539 (55.9)
City/town	5,951 (44.1)
**ADL**	
Disabled	3,104 (23)
Non-disabled	10,380 (77)
**Economic status**	
Poor/very poor	2192 (16.3)
Average	9040 (67.4)
Rich/very rich	2195 (16.3)
**Physical health status**	
Poor/very poor	2050 (16.2)
Average	4236 (33.6)
Good/very good	6331 (50.2)
**Social support**	
Poor	850 (6.3)
Good	12608 (93.7)

**Table 2:** Life satisfaction distributions.

	Life satisfaction (n, %)		
	Total Sample	Among those with concordance	Among those with discordance
**Total Sample**	7,724 (61.3%)	6,549 (63.1%)	1,171 (53.0%)
**Living arrangement**			
with next generation and spouse	981 (60.2%)	727 (62.1%)	254 (55.2%)
with next generation	4,024 (65.6%)	3,575 (66.6%)	448 (58.1%)
with spouse	1,568 (58.9%)	1,320 (59.3%)	247 (56.9%)
alone	905 (49.4%)	721 (52.8%)	182 (39.1%)
in a nursing home	246 (72.4%)	206 (79.5%)	40 (49.4%)

Note: n=12,599

**Table 3:** Adjusted ORs for the relationship between life satisfaction and living arrangements.

	Concordance (n=8,448)	Discordance (n=1,832)
With next generation VS. with next generation & spouse OR (95% CI)	0.93 (0.78∼1.11)	0.95 (0.68∼1.32)
With spouse VS. with next generation & spouse OR (95% CI)	0.79 (0.68∼0.92)*	1.45 (1.04∼2.01)*
Alone VS. with next generation & spouse OR (95% CI)	0.62 (0.53∼0.73)*	2.70 (1.74∼4.21)*
Nursing home VS. with next generation & spouse OR (95% CI)	0.84 (0.61∼1.15)	1.46 (0.74∼2.85)

Note: *represents *p<*0.05 compared with living with the next generation and a spouse
